# Editorial: Ferroptosis in cancer and Beyond—volume II

**DOI:** 10.3389/fmolb.2023.1265127

**Published:** 2023-08-29

**Authors:** Xin Wang, Jordan Lu, Guo Chen, Chaoyun Pan, Yanqing Liu

**Affiliations:** ^1^ National Institute of Neurological Disorders and Stroke, Bethesda, MD, United States; ^2^ Herbert Irving Comprehensive Cancer Center, Columbia University, New York, NY, United States; ^3^ School of Biopharmacy, China Pharmaceutical University, Nanjing, China; ^4^ Department of Biochemistry and Molecular Biology, Zhongshan School of Medicine, Sun Yat-Sen University, Guangzhou, China

**Keywords:** ferroptosis, oxidative stress, cancer, iron, disease treatment

## Introduction

It has been 11 years since Brent Stockwell identified and named ferroptosis (Dixon et al., 2012). Ferroptosis results from iron-dependent lipoxidation at various cellular membrane structures. Searching the PubMed database by using the keyword “ferroptosis” results in more than 8,000 papers. Why has ferroptosis received such intensive attention? There are at least three fundamental reasons. First, ferroptosis has a unique mechanism distinct from other known regulated cell death types. Ferroptosis is tightly associated with cell metabolism, such as amino acid, iron, and ROS metabolism. There are three key elements for ferroptosis: substrate of lipid peroxidation, executor of lipid peroxidation, and anti-ferroptosis system (Liu and Gu, 2022a). The balance between the three elements dictates the sensitivity of a cell to ferroptosis. Second, there are multiple ways to induce ferroptosis meaning it has a complex regulatory network. Many pathways are involved in ferroptosis mediation. Key factors regulating ferroptosis, including GPX4, p53, FSP1, and ALOXs have been identified (Dixon and Stockwell, 2019; Liu et al., 2019; Liu and Gu, 2022a; Liu and Gu, 2022b). However, new pathways and regulators are still emerging. Third, ferroptosis participates in the regulation of numerous physiological or pathological processes, such as normal development, degenerative diseases, ischemic injuries, immune system activities, and particularly cancer. This means that ferroptosis has amazing potential as a therapeutic target in many diseases (Stockwell et al., 2020).

To examine progress in the ferroptosis field and advances in basic research and clinical applications focusing on ferroptosis, we launched a Research Topic named Ferroptosis in Cancer and Beyond in early 2022, which was a great success. Given the rapid progression in this field, we opened a second call for this same Research Topic in late 2022, which has now been successfully closed. This second volume brings together 13 papers, including 6 research articles and 7 reviews. These articles outline recent information about ferroptosis from both basic research and clinical translation angles. The papers are briefly introduced below.

To comprehensively understand the contribution of iron regulatory proteins (IRPs) to ferroptosis, McKale Montgomery and Cameron Cardona reviewed the regulatory processes regarding iron homeostasis, from absorption, metabolism to its participation in ferroptosis, and discussed the essential roles of various IRPs in ferroptosis and their potentials to be therapeutically maneuvered in cancer treatment. To explore how ferroptosis is regulated at the post-translational level, Zhang et al. introduce emerging evidence for the O-GlcNAc modification (O-GlcNAcylation) in ferroptosis in a review article and discuss the crosstalk between O-GlcNAcylation and ROS and related antioxidant defense systems. The authors elucidate the role of O-GlcNAcylation in proteins involved in iron metabolism and the regulation of lipid metabolism and peroxidation during ferroptosis. Furthermore, the underlying mechanisms including mitochondria dysfunction and endoplasmic reticulum alteration brought by O-GlcNAcylation are discussed. In their original research study, Nikulin et al. identified *ELOVL5* and *IGFBP6* may modulate the sensitivity of breast cancer cells to ferroptosis, possibly via enhancing the activity of GPX4, an antioxidant enzyme that plays a critical role in ferroptosis. Through analysis of the transcriptomic database and validation with HPLC-MS, the knockdown of either *ELOVL5* or *IGFBP6* was shown to cause remarkable changes in the production of long and very long fatty acids. In addition, the knockdown of *ELOVL5* or *IGFBP6* in MDA-MB-231 cells promotes cell death induced by PUFAs, and the potential benefit of PUFAs addition for improving chemotherapeutic effects was proposed in the condition of low *IGFBP6* (and maybe *ELOVL5*) gene expression.

Glutathione S-transferase P1 (GSTP1) was proposed to be a potential target to tackle radioresistance in cancer therapy by Tan et al. GSTP1 is fundamental to maintaining cellular oxidative homeostasis and is involved in ferroptosis. Based on increasing evidence showing that iron metabolism, lipid peroxidation, and GSH level are modulated by radiotherapy, the authors elaborated on the potential to control GSTP1 levels to enhance the efficacy of radiotherapy in cancer treatment. More pathways in ferroptosis induced by radiotherapy and their implications for radiotherapy were reviewed by Giovanni Luca Beretta and Nadia Zaffaroni, and other strategies were proposed to improve the efficacy of radiotherapy, including enhancing ionizing radiation by other reagents or selectively inducing ferroptosis with metal-based nanoparticles. Lu et al. introduced all kinds of therapies for glioblastoma, including immunotherapy, radiotherapy, and chemotherapy, and discussed how ferroptosis participates and affects the efficacy of different therapeutic treatments. In an original research article, Shi et al. found that dihydroartemisinin (DHA), an adjuvant drug-enhancing chemotherapy, induced cervical cancer death via initiating ferroptosis and explored the involvement of ferritinophagy induced by DHA. Furthermore, DHA was also shown to have a synergistic role with doxorubicin (DOX) in promoting cervical cancer cell death.

Growing evidence has revealed the impact of T cell infiltration in the development of various types of cancer. Jiang et al. analyzed the differential gene expression in CD8^+^ T cells from CD8^+^ highly or low infiltrated samples in acute myeloid leukemia (AML) and conducted extensive bioinformatics analysis, and six ferroptosis-related genes (FRGs) were identified to generate a prognostic prediction model, which was validated to be helpful to risk stratification and prognostic prediction of AML patients. Han et al. identified several ferroptosis-related genes (FRGs) which correlate well with the immune microenvironment and establish a model to predict the prognosis of cervical cancer patients. Further mechanisms underlying iron homeostasis, ROS and lipid peroxidation, GPX4-GSH, and other regulator systems in cervical cancer were discussed in a review by Xiangyu Chang and Jinwei Miao. In another review, Lai et al. specifically elaborated on the influence of steroid hormone signaling on ferroptosis and discuss the involvement of ferroptosis in gynecologic cancers and potential therapies targeting ferroptosis for the treatment of gynecologic cancers.

With data from FerrDb and TCGA database, Li et al. established a prognostic prediction model for colorectal cancer (CRC) patients with 8 FRGs among which NOS2 is one of the most significantly affected examples and was validated with the CRC mouse model and the involvement of NF-κB pathway was elucidated. To investigate whether ferroptosis is associated with colon adenocarcinoma (COAD), Baldi et al. identified a 4-gene signature that distinguishes high-risk and low-risk patients, and those FRGs were further shown to be implicated in many pathological related pathways and a variety of miRNAs and transcription factors were found to be involved. These researches consolidated the idea that disease-associated cell death has a specific gene expression profile relevant to the prognosis of the patient (Liu et al., 2022; Ye et al., 2022; Liu et al., 2023).

Taken together, this second volume of the Research Topic Ferroptosis in Cancer and Beyond adds new knowledge to this field, furthering research and the clinical translation of ferroptosis.
